# Herbal Medicines and Ovarian Hyperstimulation Syndrome: A Retrospective Cohort Study

**DOI:** 10.1155/2016/7635185

**Published:** 2016-09-04

**Authors:** Athar Rasekhjahromi, Masoumeh Hosseinpoor, Farzaneh Alipour, Mehrnoosh Maalhagh, Saeed Sobhanian

**Affiliations:** ^1^Obstetrics and Gynecology Department, Jahrom University of Medical Sciences, Jahrom, Iran; ^2^Student Research Committee, Jahrom University of Medical Sciences, Jahrom, Iran; ^3^Student Research Committee, Shiraz University of Medical Sciences, Shiraz, Iran; ^4^Department of Community Health, Jahrom University of Medical Sciences, Jahrom, Iran

## Abstract

*Background*. The aim of this study was to assess the association between herbal medication and OHSS.* Methods*. This retrospective cohort study was conducted with 101 polycystic ovary syndrome patients. 66 patients took conventional pharmacological medications and 35 took herbal medications. Data were analyzed by statistical test including Fisher's Exact and binominal logistic regression. *P* < 0.05 was considered significant.* Results*. Of the 101 females, 53 were married and 48 were single. There was no significant association between the groups in marriage. No significant association was found in mean age between the two groups (23.9 ± 5.8 years in the control group versus 26.3 ± 6.7 years in the case group). There was a significant difference between the two groups .After adding the dependent (OHSS prevalence) and independent (marriage and group) variables into the model, the Hosmer-Lemeshow test showed suitability. Variances analyzed with this model ranged between 29.4% and 40.7%.* Conclusion*. The indiscriminate use of herbs is correlated with OHSS. Because patients increasingly consume herbs, they should be aware of potential side effects. However, appropriate dosages of herbs could be obtained for use instead of conventional treatments, which often have side effects.

## 1. Background

Polycystic ovarian syndrome (PCOS) is an endocrine disorder that is diagnosed by anovulation, hyperandrogenism, and the polycystic ovary morphology [1]. PCOS is one of the main predispositions for ovarian hyperstimulation syndrome (OHSS) [2]. OHSS is the most serious complication of ovulation induction [3]. Vasoactive products and follicle stimulating hormone (FSH) medications can also lead to OHSS [4]. OHSS is characterized as mild, moderate, or severe, based on signs, symptoms, and laboratory findings [5]. The prevalence of OHSS has increased because of the use of menotropins for controlled ovarian stimulation in assisted reproductive technologies. The incidence of OHSS from gonadotropin usage varies between 8.4% and 23% for mild OHSS, 0.005% and 7% for moderate OHSS, and 0.008% and 10% for severe OHSS [6, 7]. The symptoms of mild OHSS include abdominal distention, ovaries up to 12 cm in diameter, nausea, vomiting, and diarrhea. Moderate OHSS includes symptoms such as ascites and hydrothorax and severe OHSS includes symptoms such as rapid weight gain, severe abdominal pain, severe persistent nausea, vomiting, and hypovolemic shock [8]. The pathogenesis of OHSS is poorly understood. However, the ovarian renin-angiotensin system and secretion of an angiotensin II-like substance in the presence of high concentrations of estrogen and increased capillary permeability might play a role in OHSS development [9]. Egbase et al. hypothesized that OHSS cannot be prevented unless treatment is stopped [10].

Severe complications of OHSS are preventable. One possible factor leading to OHSS is the usage of herbal medication. Herbal remedies have been used for centuries, and their usage in both Western and Eastern society is increasing [11]. One report found that 12% of Americans, 12% of Australians, and 36.8% of patients in the UK use herbal remedies [12]. The World Health Organization estimates that up to 80% of the world's population depends on herbal medicines [13]. Although there has been concern in the medical community about possible side effects arising from herbal medicines use, very few herbal remedies have been formally evaluated [14]. The belief among patients that these products are “natural” and therefore safe is potentially dangerous. Patients who are at risk of developing OHSS should be identified before treatment because of risk factors or clinical predictors.

Both conventional pharmacological and herbal medications are used for treatment of PCOS by patients. Conventional pharmacological treatments include spironolactone, metformin, clomiphene, and gonadotropin injections [15]. Popular herbal medicines used by women in Iran include* Zingiber officinale* (ginger),* Cinnamomum verum* (cinnamon),* Thymus vulgaris* (thyme),* Carum carvi *(cumin),* Matricaria recutita *(chamomile), and* Piper nigrum *(black pepper).

Black pepper (*Piper nigrum*) has been consumed since ancient times for its anti-inflammatory and antiflatulent properties. The essential oil of pepper is composed of compounds such as piperine, an amine alkaloid, which is responsible for the herb's pungent character [16]. Ginger is commonly used to treat various types of stomach problems and pain relief in arthritis, muscle soreness, menstrual pain, and bronchitis. Ginger often has few side effects but mild side effects include heartburn, diarrhea, stomach discomfort, and extra-menstrual bleeding [17, 18]. Cinnamon is most commonly used in insulin resistance disorders. Administration of large amounts of* Cinnamomum cassia* or* Cinnamomum verum* might cause side effects in some patients. Cinnamon contains the compound coumarin, which can cause or worsen liver disease [19]. Chamomile is traditionally used for intestinal gas, motion sickness, hay fever, nervous diarrhea, attention deficit-hyperactivity disorder, and fibromyalgia. Chamomile has few side effects when taken with food, but it can cause uterine contractions that can invoke miscarriage. The National Institutes of Health recommend that pregnant women and nursing mothers should not consume chamomile [20, 21]. Thyme is often taken orally for bronchitis, whooping cough, sore throat, colic, arthritis, gastritis, diarrhea, bedwetting, dyspraxia, flatulence, and skin disorders. It is also used as a diuretic, to treat urinary tract infections, and as an appetite stimulant. Thyme is safe when consumed in food and taken as a medication for brief periods but it can cause digestive problems [22]. Cumin is used for digestive problems, heartburn, controlling urination, inducing menstruation, and increasing breast milk production [23]. We aimed to assess whether herbal medications can induce OHSS in patients with PCOS.

## 2. Methods

### 2.1. Approval

The study was initiated after approval from the research and ethics committee of Jahrom University of Medical Sciences. After clarifying the study protocol, written consent was obtained from participants.

### 2.2. Design

The retrospective cohort study was conducted based on histories of PCOS patients who used herbal remedies (*Zingiber officinale* (ginger),* Cinnamomum verum* (cinnamon),* Thymus vulgaris* (thyme),* Carum carvi *(cumin),* Matricaria recutita* (chamomile), and* Piper nigrum* (black pepper)) or conventional pharmacological treatment at a gynecology and obstetrics clinic affiliated to Jahrom University of Medical Sciences. Also patients were selected through PCOS patients. Patients who used both conventional treatment and herbal medication were excluded from the study. Patients were followed up with ultrasound to diagnose OHSS. Thirty-five patients took herbal remedies (case group), and 66 patients took pharmacological treatment (control group) by the convenience sampling method.

### 2.3. Diagnosis

Diagnosis of OHSS was based on historical and clinical findings, ultrasound, laboratory data, and physical examination. Ultrasound typically shows bilateral symmetrical enlargement of ovaries (often >12 cm in size) with multiple cysts varying in size, with a classic spoke-wheel appearance. OHSS was classified as mild, moderate, severe, or critical [24].

All patients taking herbal medicines had an ultrasonography one month before starting treatment in the obstetrics and gynecology clinic to diagnose with PCOS. After taking a history of herbal medicine usage, patients underwent another ultrasonography.

### 2.4. Statistical Analysis

The frequency of OHSS was calculated in both groups. Data were analyzed using Fisher's Exact and binominal logistic regression. *P* < 0.05 was considered significant.

## 3. Results and Discussion

Of the 101 female patients that participated in this study, 53 of the total (101) were married (71.7% of married women (number = 53) in the case group versus 28.3% of the married women (number = 53) in the control group) and 48 were single (58.3% of the single women (number = 48) in the case group versus 41.7% of the single women (number = 48) in the control group). There was no significant association between the groups in number of married patients (*P* = 0.209). No significant association was found in mean age between the groups (control group: 23.9 ± 5.8 years versus in case group: 26.4 ± 6.8 years, *P* = 0.053). There was a significant association between group and OHSS prevalence (*P* < 0.001). Three variables were placed in a stepwise binominal logistic regression model. The dependent binomial variable was prevalence of OHSS, and the independent variables were marriage status and group.

Both independent variables remained in the model. The Hosmer-Lemeshow test showed suitability with *P* = 0.401. Variances analyzed in this model ranged between 29.4% and 40.7% (Table 1).

Estimation of *β* showed that each variable might predict changes in the prevalence of OHSS. Estimations of *β* and Exp are shown in Table 2.

Herbal medications, a major component of CAM therapy, are becoming more popular. Patients often think that herbal medicine products are safe but do not fully understand their efficacy or side effects. Patients with anovulatory cycles use herbal medications in addition to their prescribed drugs but may not get the desired outcome of treatment. We performed this study to evaluate the relationship between herbal medication and OHSS. We found that herbal medication usage is correlated with OHSS prevalence. However, participants of this study had PCOS, which is one of the predisposing factors for OHSS development [6].

Wang et al. showed that cinnamon can reduce insulin resistance and might be effective in the treatment of PCOS [25]. Because higher insulin levels stimulate the ovarian response to FSH and antral follicles observed in OHSS [25], inappropriate doses of cinnamon might cause OHSS. Srivastava et al. indicated that people who are sensitive to chamomile are more prone to allergic reactions, especially if they use chamomile with other drugs [21]. Furthermore, differences in immunologic sensitivity of patients and increases in IL-18 are predictors of OHSS [26, 27].

Other trials have found correlations between Chinese herbal medication use and OHSS. Cao et al. found a higher OHSS incidence in a study on the influence of Shugan treatment on pregnancy outcome and oocyte-secreted factors in patients using* in vitro* fertilization and embryo transfer [28]. Chang et al. reported that OHSS was more common in a study on the effects of Bushentiaojing formula, a traditional Chinese medicine, on pregnancy rate and bone morphogenetic protein-15 in patients undergoing* in vitro* fertilization [29].

## 4. Conclusions

We found that indiscriminate usages of herbal medication and OHSS are correlated. Therefore, patients should pay as close attention to the side effects of natural remedies as conventional treatment. Further randomized clinical trials should be conducted to support our findings and determine safe doses of herbal medicines to avoid OHSS.

## Figures and Tables

**Table 1:** Results of the Hosmer-Lemeshow test.

Chi-square	df	Sig.
1.829	2	0.401

**Table 2:** Estimation of *β* and Exp in PCOS patients.

Exp (*β*)	*β*	S.E.	Wald	df	Sig.	Exp (*β*)	95.0% C.I. for	
							Lower	Upper
Group	2.265	0.671	11.384	1	0.001	9.628	2.583	35.882
Marriage	2.928	0.700	17.473	1	0.000	18.689	4.736	73.759
Constant	−7.035	1.830	14.780	1	0.000	0.001		

## References

[1] Norman R. J., Dewailly D., Legro R. S., Hickey T. E. (2007). Polycystic ovary syndrome. *The Lancet*.

[2] Onofriescu A., Luca A., Bors A., Holicov M., Onofriescu M., Vulpoi C. (2013). Principles of diagnosis and management in the ovarian hyperstimulation syndrome. *Current Health Sciences Journal*.

[3] Orvieto R. (2013). Ovarian hyperstimulationsyndrome—an optimal solution for an unresolvedenigma, Infertility and IVF Unit. *Journal of Ovarian Research*.

[4] Alper M. M., Smith L. P., Sills E. S. (2009). Ovarian hyperstimulation syndrome: current views on pathophysiology, risk factors, prevention, and management. *Journal of Experimental and Clinical Assisted Reproduction*.

[5] Lunenfeld B., Insler V., Glezerman M. (1993). *Diagnosis and Treatment of Functional Infertility*.

[6] Delvinge A., Rozenberg S. (2002). Epidemiology and prevention of ovarian hyperstimulation syndrome (OHSS): a review. *Human Reproduction Update*.

[7] Schenker J. G., Weinstein D. (1978). Ovarian hyperstimulation syndrome: a current survey. *Fertility and Sterility*.

[8] Budev M. M., Arroliga A. C., Falcone T. (2005). Ovarian hyperstimulation syndrome. *Critical Care Medicine*.

[9] Asch R. H., Li H.-P., Balmaceda J. P., Weckstein L. N., Stone S. C. (1991). Severe ovarian hyperstimulation syndrome in assisted reproductive technology: definition of high risk groups. *Human Reproduction*.

[10] Egbase P. E., Al Sharhan M., Grudzinskas J. G. (2002). 'Early coasting' in patients with polycystic ovarian syndrome is consistent with good clinical outcome. *Human Reproduction*.

[11] Au A. M., Ko R., Boo F. O., Hsu R., Perez G., Yang Z. (2000). Screening methods for drugs and heavy metals in Chinese Patent Medicines. *Bulletin of Environmental Contamination and Toxicology*.

[12] Skinner C. M., Rangasami J. (2002). Preoperative use of herbal medicines: a patient survey. *British Journal of Anaesthesia*.

[13] Farnsworth N. R., Wilson E. O. (1982). Rational approaches applicable to the search for and discovery of new drugs from plants. *Biodiversity*.

[14] Raynor D. K., Dickinson R., Knapp P., Long A. F., Nicolson D. J. (2011). Buyer beware? Does the information provided with herbal products available over the counter enable safe use?. *BMC Medicine*.

[15] Barbieri R. L., Nabel E. G. (2010). Polycystic ovary syndrome. *ACP Medicine*.

[16] Singh A., Rao A. R. (1993). Evaluation of the modulatory influence of black pepper (*Piper nigrum*, L.) on the hepatic detoxication system. *Cancer Letters*.

[17] Vutyavanich T., Kraisarin T., Ruangsri R.-A. (2001). Ginger for nausea and vomiting in pregnancy: randomized, double-masked, placebo-controlled trial. *Obstetrics and Gynecology*.

[18] Terry R., Posadzki P., Watson L. K., Ernst E. (2011). The use of ginger (*Zingiber officinale*) for the treatment of pain: a systematic review of clinical trials. *Pain Medicine*.

[19] Baker W. L., Gutierrez-Williams G., White C. M., Kluger J., Coleman C. I. (2008). Effect of cinnamon on glucose control and lipid parameters. *Diabetes Care*.

[20] Habersang S., Leuschner F., Isaac O., Thiemer K. (1979). Pharmacological studies with compounds of chamomile: IV. Studies on toxicity of (−)-*α*-Bisabolol. *Planta Medica*.

[21] Srivastava J. K., Shankar E., Gupta S. (2010). Chamomile: a herbal medicine of the past with a bright future. *Molecular Medicine Reports*.

[22] Kohlert C., Schindler G., März R. W. (2002). Systemic availability and pharmacokinetics of thymol in humans. *Journal of Clinical Pharmacology*.

[23] May B., Köhler S., Schneider B. (2000). Efficacy and tolerability of a fixed combination of peppermint oil and caraway oil in patients suffering from functional dyspepsia. *Alimentary Pharmacology and Therapeutics*.

[24] Smits G., Olatunbosun O., Delbaere A., Pierson R., Vassart G., Costagliola S. (2003). Ovarian hyperstimulation syndrome due to a mutation in the follicle-stimulating hormone receptor. *New England Journal of Medicine*.

[25] Wang J. G., Anderson R. A., Graham G. M. (2007). The effect of cinnamon extract on insulin resistance parameters in polycystic ovary syndrome: a pilot study. *Fertility and Sterility*.

[26] Gardner D. K., Rizk B. R. M., Falcone T. (2011). *Human Assisted Reproductive Technology: Future Trends in Laboratory and Clinical Practice*.

[27] Barak V., Elchalal U., Edelstein M., Kalickman I., Lewin A., Abramov Y. (2004). Interleukin-18 levels correlate with severe ovarian hyperstimulation syndrome. *Fertility and Sterility*.

[28] Cao H., Han M., Ng E. H. Y. (2013). Can Chinese herbal medicine improve outcomes of in vitro fertilization? A systematic review and meta-analysis of randomized controlled trials. *PLoS ONE*.

[29] Chang X. F., Du H. L., Gao X., Zhang M., Zhang J. P., Zhu A.-P. (2011). Effects of Bushentiaojing formula (traditional Chinese medicine) on pregnancy rate and bone morphogenetic protein-15 in patients undergoing in vitro fertilization. *Reproduction and Contraception*.

